# IRES-dependent translation of the long non coding RNA *meloe* in melanoma cells produces the most immunogenic MELOE antigens

**DOI:** 10.18632/oncotarget.10923

**Published:** 2016-07-29

**Authors:** Maud Charpentier, Mikael Croyal, Delphine Carbonnelle, Agnès Fortun, Laetitia Florenceau, Catherine Rabu, Michel Krempf, Nathalie Labarrière, François Lang

**Affiliations:** ^1^ CRCNA, INSERM, CNRS, Université d'Angers, Université de Nantes, Nantes, France; ^2^ UMR INRA 1280, CHU, Nantes, France; ^3^ West Human Nutrition Research Center, CHU, Nantes, France; ^4^ CHU, Nantes, France

**Keywords:** melanoma, IRES, long non coding RNA, tumor antigens, immunotherapy

## Abstract

MELOE-1 and MELOE-2, two highly specific melanoma antigens involved in T cell immunosurveillance are produced by IRES-dependent translation of the long « non coding » and polycistronic RNA, *meloe*. In the present study, we document the expression of an additional ORF, MELOE-3, located in the 5′ region of *meloe*. Data from *in vitro* translation experiments and transfection of melanoma cells with bicistronic vectors documented that MELOE-3 is exclusively translated by the classical cap-dependent pathway. Using a sensitive tandem mass spectrometry technique, we detected the presence of MELOE-3 in total lysates of both melanoma cells and normal melanocytes. This contrasts with our previous observation of the melanoma-restricted expression of MELOE-1 and MELOE-2. Furthermore, *in vitro* stimulation of PBMC from 6 healthy donors with overlapping peptides from MELOE-1 or MELOE-3 revealed a very scarce MELOE-3 specific T cell repertoire as compared to the abundant repertoire observed against MELOE-1. The poor immunogenicity of MELOE-3 and its expression in melanocytes is consistent with an immune tolerance towards a physiologically expressed protein. In contrast, melanoma-restricted expression of IRES-dependent MELOE-1 may explain its high immunogenicity. In conclusion, within the MELOE family, IRES-dependent antigens represent the best T cell targets for immunotherapy of melanoma.

## INTRODUCTION

In the field of cancer immunotherapy, the recently described effectiveness of checkpoint inhibitors such as anti-PD1 blocking antibodies to boost anti-tumor T cell responses is very encouraging [1]. However, a significant number of patients are non responders to these therapies and thus, there is still room for improvement using antigen-specific immunotherapy, whether through vaccination or through T cell transfer. In this respect, the choice of the targeted antigens remains a critical issue [2] and the ideal antigen(s) should have the following characteristics: be as tumor specific as possible and stimulate a broad T cell repertoire in the majority of patients (i.e. generate many epitopes in various HLA contexts). In this respect, we have previously identified two melanoma antigens, namely MELOE-1 and MELOE-2, that were recognized by tumor-infiltrating lymphocytes (TIL) from HLA-A0201+ patients who remained relapse-free following TIL transfer in an adjuvant setting [3].

Unexpectedly, these two antigens were translated from a single unspliced RNA that we named *meloe* since this RNA was overexpressed in the melanocytic lineage. This RNA probably belongs to the family of long intronic non coding RNA (lncRNA) [4] since it shares many of their features: it is located in the intron of HDAC4 in antisense direction, it is capped and polyadenylated and contains no long ORF but multiple short ORFs (< 100 aa) and is transcribed in a tissue specific manner i.e. the melanocytic lineage [5, 6]. Despite their denomination as « non coding » RNAs, it was shown that many lncRNAs can in fact be translated into short polypeptides [7, 8, 9].

In the case of *meloe*, we demonstrated that the translation of the MELOE-1 and 2 polypeptides (39 aa and 46 aa long respectively) in melanoma cell lines was achieved by an IRES-dependent mechanism [10]. In contrast, although normal melanocytes expressed *meloe* RNA, they were not recognized by MELOE-1 or MELOE-2 specific T cell clones suggesting that MELOE-1 and 2 were not translated in these cells.

We also provided evidence that a broad T cell repertoire against the MELOE-1/HLA-A2 epitope was present in both melanoma patients and healthy individuals [11] and that processing of MELOE-1 could also generate several class II epitopes in various HLA contexts [12, 13].

Since *meloe* RNA contains many other ORFs close to the 5′ end, we wondered whether this RNA could also generate polypeptide(s) through cap-dependent translation and whether this new MELOE polypeptide(s) would be immunogenic. In the present report, we identify a new polypeptide of the MELOE family, MELOE-3, and describe its expression and its immunogenicity in comparison with that of MELOE-1 to evaluate its potential value as a T cell target for melanoma immunotherapy.

## RESULTS

### A new ORF from *meloe* RNA is efficiently translated in melanoma cells

In the course of precisely defining the +1 transcription start of the *meloe* RNA, we have previously shown that the transcript is in fact 259 bp longer at the 5′ end than the public sequence reported in the NCBI data bank [NR-026664] [5]. Within this added sequence, three putative ORFs are present and we focused our attention on ORF_132-296_ (Supplementary Figure S1) because it contained the best initiation sequence (AUGG) and would code for a 54 aa long polypeptide, coined MELOE-3. To check whether this ORF could be translated from *meloe* RNA in melanoma cells, we transfected the melanoma cell line M113 with a construct composed of the full length *meloe* RNA in which this ORF was replaced by a sequence coding for eGFP-MELOE-3 (Supplementary Figure S2) and compared it to M113 transfected with the previously described eGFP-MELOE-1 construct or with the native *meloe* cDNA as a negative control [10].

**Figure 1:**
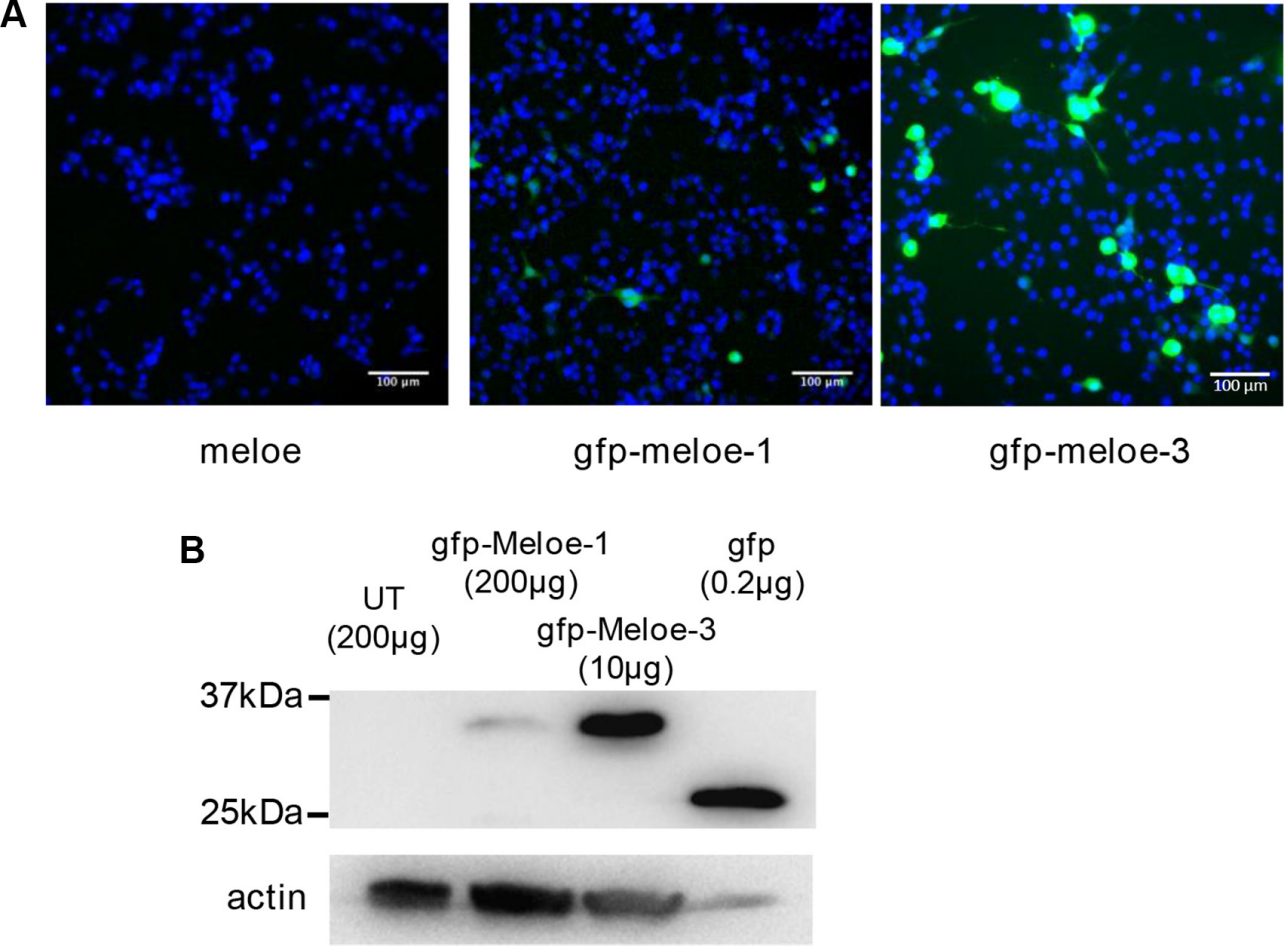
Expression of MELOE-1 and MELOE-3 in melanoma cells (**A**) M113 melanoma cells were transfected with *meloe*-eGFP constructs: the native *melo*e cDNA (left panel), eGFP-MELOE-1 (middle panel) and eGFP-MELOE-3 (right panel). Analysis of fluorescent cells 48 h post-transfection was made using an automated fluorescence High Content Screening (HCS) microscopic device. Representative images out of 49 scanned fields per condition are shown. Nuclei were Hoescht stained prior to analysis. (**B**) Fluorescent eGFP-MELOE-1 (lane 2) or eGFP-MELOE-3 (lane 3) proteins were detected by western blot using an anti-eGFP mAb. Untransfected (UT) cells (lane 1) or cells transfected with the peGFP-N3 plasmid (lane 4) were used as controls. Amounts of total proteins loaded in each lane are indicated. A typical experiment is presented out of 4 performed.

As shown on Figure 1A in a typical experiment, the percentage of fluorescent melanoma cells detected with an HCS array scan reader was much higher following transfection with the eGFP-MELOE-3 construct than with the eGFP-MELOE-1 construct (29.5% for MELOE-3 vs 4.8% for MELOE-1). Similar percentages were obtained in two other experiments that were also confirmed by flow cytometry (data not shown). Moreover, the higher intensity of fluorescence of positive cells with eGFP-MELOE-3 suggested a more efficient translation than that of the eGFP-MELOE-1 construct.

To confirm that these observed differences in fluorescence reflected differences in amounts of translated protein, we performed a Western blot analysis of lysates of M113 cells transfected with the two constructs using an anti-eGFP monoclonal antibody. Untransfected cells and eGFP-transfected cells were used as negative and positive control respectively. As shown on Figure 1B, the expression of eGFP-MELOE-3 was much higher than that of eGFP-MELOE-1, all the more as only 10 μg of protein lysate of eGFP-MELOE-3-transfected cells were loaded as compared to 200 μg of lysate of eGFP-MELOE-1 transfected cells. These data strongly suggested that MELOE-3 could be very efficiently translated from *meloe* RNA in melanoma cells. Considering its location close to the 5′ end of the transcript, we were prompted to test whether the translation of this ORF would be cap-dependent.

### MELOE-3 is translated by a classical cap-dependent mechanism

**Figure 2:**
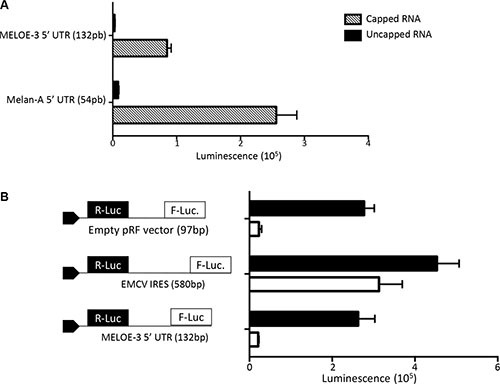
MELOE-3 translation mechanism (**A**) *In vitro* transcribed RNA containing the Melan-A 5′UTR (positive control) or the MELOE-3 5′UTR upstream of Firefly luciferase with or without addition of a 5′MeG were *in vitro* translated using the Rabbit reticulocyte system. Luminescence is expressed in arbitrary units. (**B**) pRF bicistronic vectors containing the 5′UTR of MELOE-3, the viral IRES EMCV (positive control) or empty (control plasmid) were transfected into M113 melanoma cell line. Renilla (black bars) and Firefly (white bars) luciferase activities were measured after 48 h and expressed in arbitrary units. Data are expressed as mean ± SD from three different experiments.

To assess whether MELOE-3 translation was cap-dependent or not, we used an *in vitro* transcription and translation assay. We designed RNA constructs comprising either the 5′end of *meloe* upstream of MELOE-3 (1–132 bp) or the 5′UTR of Melan-A (54 bp) coupled to the Firefly luciferase coding sequence. Each construct was either capped or uncapped and used as translation templates in the rabbit reticulocyte lysate system. As shown on Figure 2A, the capped Melan-A construct, used as positive control, was very efficiently translated resulting in high firefly luminescence while this translation was absent with the uncapped construct. Likewise, only the capped MELOE-3 construct was translated demonstrating that the cap was compulsory for efficient translation in this system.

In addition, to confirm that no other translation mechanism could be involved in the expression of MELOE-3, we checked for the presence of IRES activity in the same upstream region of MELOE-3 in a cellular assay. We used bicistronic Renilla/Firefly expression vectors in which we cloned either the sequence of the EMCV IRES sequence as positive control or the 5′end of *meloe* and transfected M113 melanoma cells with them as previously described [10]. In 3 distinct experiments, we could not detect any IRES activity within the upstream region of MELOE-3 while the EMCV IRES sequence allowed efficient translation of firefly luciferase as expected (Figure 2B). These data further supported the hypothesis that MELOE-3 is exclusively translated by a classical cap-dependent mechanism in melanoma cells.

### MELOE-3 is expressed in both melanoma cells and normal melanocytes

A few years ago, after we had identified MELOE-1 as a source of T cell epitopes in melanoma cells, we had a monoclonal antibody made against MELOE-1, whose specificity and affinity was confirmed by ELISA on synthetic MELOE-1. However, despite our efforts, we could not detect the whole MELOE-1 polypeptide in melanoma cell lines by Western blot or by flow cytometry with this antibody (data not shown). We reasoned that this may be due to too low levels of MELOE-1 translation and/or the short half-life of the polypeptide that may be quickly degraded by the proteasome to generate T cell epitopes. In contrast, MELOE-3 seemed to be translated in much greater amounts according to the transfection experiments mentioned above and we figured that it may be possible to detect it in untransfected melanoma cells with a specific antibody. Moreover, we reasoned that since the *meloe* transcript is present in normal melanocytes, cap-dependent translation of MELOE-3 should also occur in normal melanocytes.

**Figure 3:**
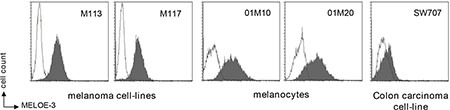
MELOE-3 staining Melanocytes (01M10, 01M20), melanoma cell lines (M113, M117) and one colon carcinoma cell-line (SW707) were stained with a custom-made MELOE-3 monoclonal antibody (75 μg/mL) and a PE-coupled anti-mouse F(ab')_2_ (dark histograms) and analyzed by flow cytometry. A mouse control isotype was used as negative control (clear histograms).

We thus had a monoclonal antibody made against MELOE-3 and used it to screen melanoma cell lines, normal melanocytes and the colon carcinoma cell line SW707 as negative control by flow cytometry on fixed and permeabilized cells. As shown on Figure 3, both normal melanocytes and melanoma cell lines were stained with the antibody while only a weak staining was observed on the SW707 cell line. However, our attempts to visualize MELOE-3 by Western blot with or without previous immuno-precipitation were unsuccessful, possibly in part because our mAb had a low affinity (10^–6^ M) for MELOE-3.

**Figure 4:**
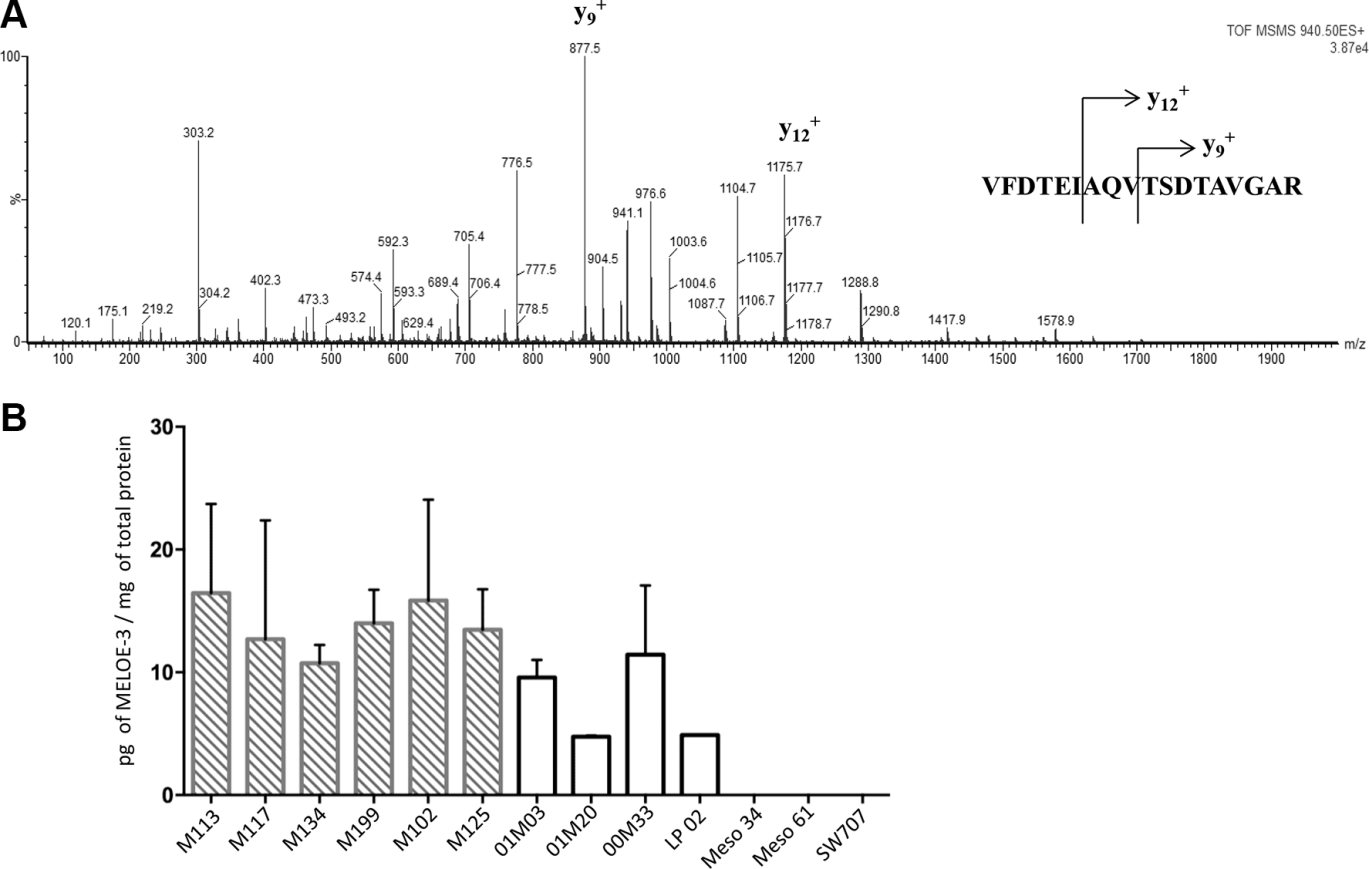
Detection and quantification of MELOE-3 by mass spectrometry (**A**) The full length MELOE-3 synthetic peptide was trypsin digested and the VFDTEIAQVTSDTAVGAR (m/z 940.5) sub peptide signature was isolated and fractionated into 29 product ions by tandem mass spectrometry. The major MS/MS transitions 940.5 → 877.7 (y_9_^+^; TSDTAVGAR) and 940.5 → 1175.5 (y_12_^+^; AQVTSDTAVGAR) transitions were selected as MELOE-3 signatures. (**B**) Cell lysates from 6 melanoma cell lines (hatched bars) and 4 melanocytes cell lines (white bars) were trypsin digested and MELOE-3 transitions 940.5 → 877.7 and 940.5 → 1175.5 were detected by LC-MS/MS. A standard curve with a range of concentrations of synthetic peptides was used for quantification (see M&M and Supplementary Figure S3). Mesothelioma cell lines (Meso 4, Meso 61) and a colon carcinoma cell line (SW707) were used as negative controls. Data are mean ± SD from 3 distinct experiments.

We thus decided to use LC-MS/MS to formally document the presence of MELOE-3 in melanocytes and melanoma cell lines. Digestion of MELOE-3 synthetic peptide by trypsin produced three peptide fragments among which the VFDTEIAQVTSDTAVGAR peptide had an appropriate size for subsequent LC-MS/MS analysis. The MS/MS fragmentation patterns of this peptide gave 29 transitions allowing us to fully sequence and thus unambiguously identify it (Figure 4A). We chose two major and specific MS/MS transitions for MRM detection, 940.5 → 877.7 (y_9_^+^; TSDTAVGAR) and 940.5 → 1175.5 (y_12_^+^; AQVTSDTAVGAR) as MELOE-3 signatures to assess its presence in different cell types. Since both transitions gave peaks of comparable intensities, instead of choosing one or the other, both transitions were summed for quantification. Total cell lysates from 6 different melanoma cell-lines and 4 melanocytes cultures were subjected to trypsin digestion and injected into the LC-MS/MS analyzer. Lysates from 2 mesothelioma cell lines and 1 colon carcinoma cell line in which *meloe* transcription is respectively absent or very low [5] were used as negative controls. Using this very sensitive and specific technique, MELOE-3 was detected in total lysates from all melanocytes cultures and melanoma cell lines but not in mesothelioma or colon carcinoma cell lines (Figure 4B). Quantification in cell lysates revealed that some normal melanocytes could express similar amounts of MELOE-3 than some melanoma cell lines (Figure 4B). As a whole, melanoma cell-lines expressed higher levels of MELOE-3 than melanocytes (13.9 ± 5.0 pg/mg of protein vs 8.1 ± 3.9 pg/mg, *p* = 0.02).

### MELOE-3 is a poor immunogen as compared to MELOE-1

**Figure 5:**
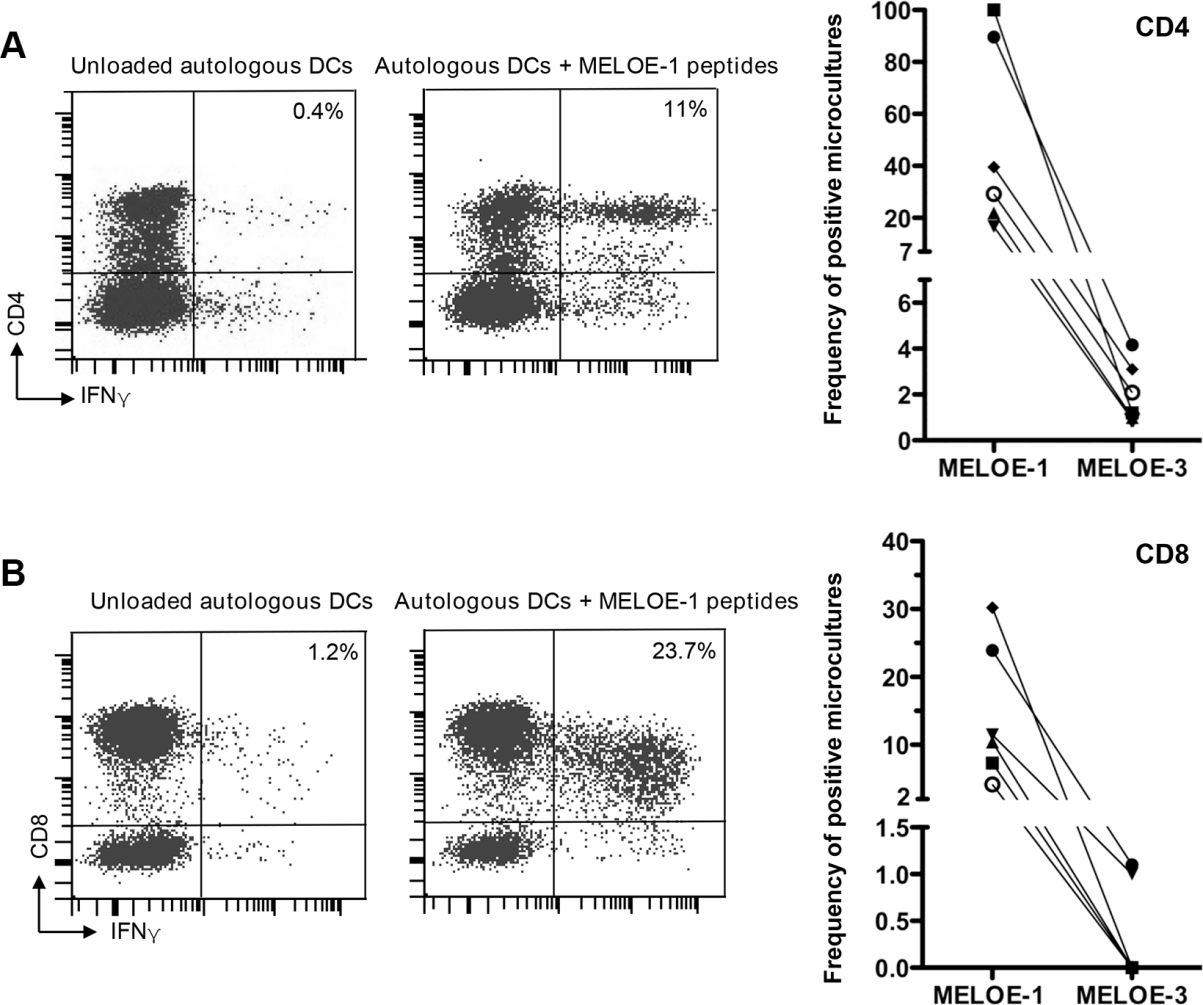
MELOE-1 and MELOE-3 immunogenicity PBMCs from 6 healthy donors were primed for 25 days with overlapping 15 or 20 aa peptides from MELOE-1 or MELOE-3 in the presence of a cytokines mix designed to accelerate DC differentiation and maturation (acDCs) (see M&M). After restimulation with autologous acDCs loaded with MELOE-1 or MELOE-3 peptides, microcultures were screened for CD4^+^ IFNγ+ (panel **A**) and CD8^+^ IFNγ^+^ (panel **B**). Unloaded acDCs were used as negative control. Examples of positive CD4 or CD8 responses are shown on the left panels and summary of responses detected in the 6 donors is shown on the right. The threshold of positivity for a microculture was set at 0.5% INFγ^+^ producing T cells after substraction of background.

We have previously shown that a broad and frequent T cell repertoire against MELOE-1 epitopes is present in melanoma patients and also in healthy individuals [11] and we wondered whether this would be the case for MELOE-3. To evaluate its immunogenicity in comparison with that of MELOE-1, we performed *in vitro* peptide stimulations of PBMC from 6 HLA-A*0201 healthy donors using 15–20 mers overlapping peptides covering the entire sequence of either MELOE-1 or MELOE-3 (Supplementary Figure S4). We used a mix of cytokines previously described to favor fast DC differentiation and maturation within total PBMC [15] and after 25 days of culture, we rechallenged the 96 initial microcultures with autologous monocytes-derived DC loaded or not with the same overlapping peptides. The presence of specific T cell responses was evaluated by INFγ intracellular staining coupled to CD4 or CD8 T cell staining from each microculture. The threshold of positivity for a microculture was set at 0.5% INFγ ^+^ producing T cells after subtraction of the background production against unloaded DC (Figure 5).

The reason for selecting HLA-A*0201 individuals was that it allowed us to check in parallel the CD8 response towards the HLA-A*0201 epitope from MELOE-1 by tetramer staining as an internal control. This selection of HLA-A*0201 donors did not bias *a priori* the stimulation in favor of MELOE-1 responses since MELOE-3 also contains putative HLA-A*0201 epitopes (www-bimas.cit.nih.gov) (in red in Supplementary Figure S4).

On Figure 5 is shown the percentage of microcultures containing CD4 specific T cells (panel A) and CD8 specific T cells (panel B) against MELOE-1 and MELOE-3 for each of the 6 donors. In agreement with our previous observations, all 6 healthy donors displayed a high percentages of CD4 T cell responses against MELOE-1 (range 16/96 to 96/96 positive microcultures). In marked contrast, CD4 responses against MELOE-3 were very scarce: 3 donors had only 1/96 positive culture and the other 3 donors had 2/96, 3/96 and 4/96 positive cultures respectively (*p* = 0.02 for MELOE-1 vs MELOE-3 responses). Likewise, a marked difference in CD8 T cell reactivity against MELOE-1 and MELOE-3 was observed: all 6 donors displayed significant CD8 T cell responses against MELOE-1 (range 4/96 to 29/96 positive cultures) that were confirmed by tetramer staining (data not shown) while CD8 reactivity against MELOE-3 was absent in 4 donors and detectable in only 1/96 culture in the 2 other donors (*p* = 0.009 for MELOE-1 vs MELOE-3 responses).

## DISCUSSION

A number of publications have suggested an important role of long non coding (lnc) RNAs in regulating gene expression through various mechanisms including epigenetic modifications (for review, [4]). In addition, over-expression of some lncRNA evidenced by RNAseq has been implicated in cancer progression in many different cancer types while other lncRNA act as tumor suppressors [18]. Aside from their role as gene expression regulators, lnc RNA have ribosome profiling signatures consistent with translation [8, 9] and we hypothesize that they may represent a good source of new immunogenic peptides to target in cancer immunotherapy. In fact, the lnc RNA *meloe* was identified by screening a melanoma cDNA library in search for the antigens recognized by tumor infiltrating T lymphocytes from a melanoma patient [3]. Following the identification of MELOE-1 and MELOE-2 antigens translated from two distinct ORFs of *meloe* in an IRES-dependent manner and considering their relatively low levels of expression even after transfection of *meloe* into melanoma cells [10], we wondered whether other ORFs closer to the 5′end of the RNA may be more efficiently translated. We selected the ORF_132-296_ since it codes for a putative 54 aa long peptide that would be long enough to contain potential class I and class II epitopes. Data obtained with melanoma cell lines transfected with the eGFP-MELOE-3 construct demonstrated that this ORF could be very efficiently translated, with amounts of translated chimeric protein that were much higher than that obtained after transfection with the IRES-dependent eGFP-MELOE-1 construct. Our *in vitro* translation experiments documented the cap dependency of MELOE-3 expression and transfection experiments with bicistronic constructs demonstrated the absence of IRES activity upstream of this ORF. We reasoned that this higher level of expression as compared to MELOE-1 should also occur in untransfected cells and may allow us to detect the native MELOE-3 protein in melanoma cell lines by monoclonal antibody staining. We had a MELOE-3 mAb made and indeed we detected a specific staining in melanoma cell-lines and in melanocytes but not in the colon carcinoma cell-line SW707. However with this MELOE-3 mAb, we were unable to visualize native MELOE-3 in cell lysates by immunoprecipitation and Western blot. Therefore, to formally document the presence of native MELOE-3 in melanocytes and melanoma cells, we used the very sensitive and specific LC-MS/MS technique [16, 17] that allowed unambiguous identification and quantification of MELOE-3 within a complex mixture of proteins in the lysates. Since melanocytes and melanoma cell-lines expressed variable but comparable levels of *meloe* RNA [5] it was not unexpected that levels of the cap-dependent MELOE-3 protein would also be comparable in the two types of cells. Nevertheless, this feature is in marked contrast with the IRES-dependent expression of MELOE-1 and MELOE-2 which was restricted to melanoma cell lines as assessed by specific T cell clone recognition [3, 19]. In the case of MELOE-1 and MELOE-2, we strongly suspect that their translation is restricted to melanoma cells because their IRES-transactivating factors (ITAF) are exclusively activated during the transformation process. In fact, a number of publications have reported activation of IRES-dependent translation of proteins that may contribute to tumorigenesis, metastasis or survival in various cancer cells [20–22, 23]. Moreover, it was previously reported that IRES translation could lead to the expression of a novel protein, MPD6, in cancer cells (prostate cancer, chronic myelogenous leukemia) distinct from that coded by the main ORF (myotrophin) and generate a strong immune response [24]. We hypothesize that, likewise, the strong immunogenicity of MELOE-1 and 2 results from their IRES-dependent melanoma specific expression and thus their avoidance of immune tolerance.

To challenge this hypothesis, we explored whether MELOE-3, the new member of the MELOE family translated from the same *meloe* RNA but through a classical cap-dependent process would be immunogenic. In the six healthy individuals tested, both CD4 and CD8 responses against MELOE-3 were very significantly reduced as compared to responses against MELOE-1. Those results are in agreement with our previous unsuccessful attempts to generate MELOE-3 specific CD4 or CD8 T cell clones by repeated *in vitro* stimulations of PBMC from 3 healthy donors and two melanoma patients with DC loaded with MELOE-3 full length or with overlapping peptides (unpublished results).

Considering the relative high level of expression of MELOE-3 compared to that of MELOE-1, these low frequencies of CD4 and CD8 reactive T cells against MELOE-3 suggest an immune tolerance against this protein that could result from its expression in normal melanocytes. These data thus support the hypothesis of a correlation between IRES-dependency, melanoma-restricted expression and immunogenicity.

In conclusion, our data strongly suggest that, within the family of MELOE antigens, IRES-dependent antigens are the most immunogenic and represent the best targets for immunotherapy in melanoma. Considering the wealth of recently identified cap-independent translated sequences [25], we are prompted to explore whether this concept may be extended to other IRES-dependent tumor antigens.

## MATERIALS AND METHODS

### Cell lines and PBMC

Melanoma cell lines were established from fragments of metastatic tumors and registered in the Biocollection PC-U892-NL (CHU Nantes). Human Mesothelioma cell lines, Meso34 and Meso61 belonging to the Biocollection PCU892-MG were gifts from Dr. Grégoire (INSERM U892, Nantes, France). The colon carcinoma cell line SW707 was purchased from ATCC (Manassas, USA). Cell lines were grown in RPMI 1640 containing 10% fetal calf serum (FCS) (Sigma, Lyon, France), 2 nM L-glutamine, 100 UI/mL penicillin and 0.1 mg/mL streptomycin (Gibco). Human melanocytes (01M03, 01M20, 01M10 and 00M33) were gifts from M. Regnier (L'Oréal Laboratory, Paris, France) or purchased from Life Technologies (St-Aubin, France) (LP-02). Melanocytes were cultured in supplemented medium 254 HMGS (Life Technologies). PBMC were isolated from healthy HLA-A*0201 donors (Etablissement Français du Sang, Nantes, France).

### Construction of plasmids

We constructed chimeric cDNAs comprizing the eGFP cDNA fused to the *meloe* ORF coding for MELOE-1 or MELOE-3 and replaced the original ORF with these constructions within the full length *meloe* cDNA as previously described [10] (Supplementary Figure S2). These modified *meloe*-eGFP cDNAs were cloned into the pCDNA3 expression vector (Invitrogen, Life Technologies) and transiently transfected into melanoma cells. For bicistronic constructs the 5′UTR region of MELOE-3 or the EMCV viral IRES used as positive control was cloned into the pRF vector between Renilla and Firefly luciferase ORFs as previously described [14]. Monocistronic plasmids harbouring either the 5′UTR region of MELOE-3 or the 5′UTR region of Melan-A upstream to the Firefly luciferase were constructed into the pGL4 vector (Promega, Charbonnières, France) to assess cap dependency of the translation.

### Transient transfections

Melanoma cell lines grown at 50–70% confluency were transfected with 10 μg eGFP reporter plasmid/106 cells and LTX lipofectamine (Invitrogen) according to the manufacturer's instructions. eGFP fluorescence was analysed 48 h post-transfection using the Image Stream^®^ Mark II Imaging flow cytometer (Merck Millipore, Darmstadt, Allemagne) or an automated fluorescence High Content Screening (HCS) microscopic device (Array Scan VTI, thermo Scientific, Courtaboeuf, France). Acquisition of Hoescht stained nuclei and eGFP expressing cells was made using a 386/420 nm and 485/515 nm excitation/emission filters with a 10X objective. Forty-nine fields per well were analysed with Cellomics^®^ View Software (Thermo Fisher Scientific). For luciferase reporter assays, cells were lysed 48 h post-transfection and luminescence was measured on a VICTOR X3 apparatus (Perkin Elmer, Courtaboeuf, France). Luminescence is expressed in arbitrary units.

### *In vitro* transcription and translation

Monocistronic constructs under a T7 promoter were linearized with XhoI and transcribed using mMessage mMachine^®^ T7 kit (Ambion^®^ Thermo Fischer Scientific, Waltham, USA) for capped RNAs and the T7 RiboMAX^™^ Express kit (Promega, Fitchburg, USA) for uncapped RNAs. Poly-A tailing was performed afterwards using the poly-A tailing kit (Ambion^®^). *In vitro* translation was performed according to the guidelines of the manufacturer using Rabbit Reticulocyte Lysate (RRL) (Promega, Madison, USA) on 100 ng of capped or uncapped RNA. Luminescence was measured with a VICTOR X3 and expressed in arbitrary units.

### Western blots

Cells were lysed 48 h post-transfection with HEPES pH3.7 30 mM, KCl 160 mM, MgCl2 2.5 mM, DTT 1 mM, NP-40 0.1%, Triton X100 0.5%, glycerol 10% with protease inhibitors (Roche, Boulogne-Billancourt, France). Protein content was quantified by a BC Assay (Interchim, Montluçon, France). Whole protein samples were run on a 12% SDS-PAGE gel and blotted onto Immobilon^®^-P PVDF membranes (Millipore, Molsheim, France). Membranes were stained overnight with an eGFP Ab (1 μg/mL) (Clontech, Mountain View, USA). After hybridization with a HRP-conjugated secondary antibody ECL detection (BioRad, Marnes-la-Coquette, France) was performed. Staining was analysed with ChemiDoc^™^ MP Imaging system (BioRad). Actin was used as a loading control (MAB1501, Millipore). Images were adjusted for contrast and brightness using the Image J software.

### Flow cytometry

Cell lines were stained with a custom made MELOE-3 mAb (75 μg/mL; Proteogenix, Schiltigheim, France) and a PE coupled Anti-Mouse IgG (Fcgamma) (500 ng/mL; Beckman Coulter, Pasadena, USA) as secondary antibody. A mouse IgG1 isotype (75 μg/mL; Abcam, Cambridge, UK) was used as negative control.

### Stimulation of PBMC with MELOE-1 and MELOE-3 peptides

At day 0, PBMCs were plated in 96 well-plates at 2 × 10^5^ cells/wells in RMPI 1640 medium containing 8% human serum, 50 UI/mL IL-2 (Proleukin, Novartis,) and stimulated with 10 μM of overlapping MELOE-1 or MELOE-3 peptides (Supplementary Figure S2) purchased from Proteogenix. DC differentiation and maturation was performed by addition of 1000 U/mL of GM-CSF (CellGenix, Freiburg, Germany) and 500 UI/mL of IL-4 (CellGenix). After 24 hours, TNFα (1000 UI/mL), IL-1Δ (10 ng/mL) and prostaglandin E_2_ (1 μM) (R&D Systems, Minneapolis, USA) were added according to the « accelerated monocyte derived dendritic cells » (acDCs) protocol [15]. After 25 days, PBMCs were re-stimulated with acDCs loaded with overlapping MELOE-1 or MELOE-3 peptides for 5 h in presence of 10 μg/mL Brefeldin A (Sigma). The percentage of microcultures containing specific CD4+ and CD8+ T cells was evaluated by surface staining with anti-CD8 mAb (clone RPA-T8, BioLegend, San Diego, USA) or anti-CD4 mAb (clone RPA-T4, BioLegend) and intracellular staining with an anti-INFγ mAb (clone 45–15, Miltenyi Biotec, Paris, France) after 4% paraformaldehyde fixation and 0.1% saponin permeabilisation.

### LC-MS/MS analysis of cell lysates

MELOE-3 was sought in cell lysates by liquid chromatography-tandem mass spectrometry (LC-MS/MS), a very sensitive method that we previously used to identify specific peptides within complex protein mixtures [16, 17].

All reagents were obtained from Sigma Aldrich (Saint-Quentin Fallavier, France) and synthetic VFDTEIAQVTSDTAVGAR and VFDTEIAQVTSDTAVGA-[^13^C_6_, ^15^N_4_]R peptides were purchased from Thermo Scientific Biopolymers (Einsteinstrasse, Germany). Cell lysates were mixed with 50 mM ammonium bicarbonate buffer (pH 8) containing 100 nM of VFDTEIAQVTSDTAVGA-[^13^C_6_, ^15^N_4_]-R as internal standard, 10% aqueous sodium deoxycholate and 500 mM of aqueous dithiothreitol. The samples were reduced for 30 min at 60°C, then alkylated with fresh iodoacetamide solution (1 M in 1 M sodium hydroxide solution) for 60 min at room temperature, and protected from light. The samples were digested overnight with a 0.1 mg/mL trypsin solution in 1 mM hydrochloric acid. Formic acid (20%) was added to stop the reaction. Finally, samples were centrifuged at 15,000 × g and 4°C for 15 min, and the supernatants were transferred to vials for LC/MS/MS analyses.

Analyses were performed on a Xevo^®^ Triple-Quadrupole mass spectrometer with an electrospray (ESI) interface equipped with an Acquity H-Class^®^ UPLCTM device (Waters Corporation, Milford, MA, USA). Labeled and unlabeled peptides were separated on an Acquity^®^ BEH C_18_ column (2.1 × 100 mm, 1.7 μm, Waters) at 60°C with a linear gradient of mobile phase B (acetonitrile containing 0.1% formic acid) in mobile phase A (5% acetonitrile in water containing 0.1% formic acid) and at a flow rate of 600 μL/min. Mobile phase B was linearly increased from 1% to 50% for 5 min, kept constant for 1 min, returned to the initial condition over 1 min, and kept constant for 1 min before the next injection. Ten microliters of each sample were injected into the LC column. Labeled and unlabeled peptides were then detected by the mass spectrometer with the ESI interface operating in the positive ion mode (capillary voltage, 3 kV; desolvation gas (N2) flow and temperature, 1000 L/h and 400°C; source temperature, 120°C). The multiple reaction monitoring (MRM) mode was applied for MS/MS detection (VFDTEIAQVTSDTAVGAR: m/z 940.5 → 1175.5 + 877.7; VFDTEIAQVTSDTAVGA-[13C6,15N4]R: m/z 945.5 → 1185.5 + 887.7) with cone and collision set at 55 and 40 V, respectively. Chromatographic peak area ratios between unlabeled and labeled peptides constituted the detector responses. Standard solutions were used to plot calibration curves and a linear regression model (1/× weighting) was used for quantification.

### Statistical analysis

Data are expressed as mean ± SD. Statistical analyses were performed to compare the percentage of MELOE-1 and MELOE-3 specific INFγ positive micro-cultures after sensitization with the corresponding peptides. Normal distribution was evaluated by a Kolmogorov-Smirnov normality test and a paired *t* test was used to compare the percentage of CD4+ or CD8+ IFNγ positive microcultures in each donor. Statistical analyses were performed using PRISM software.

## SUPPLEMENTARY MATERIALS FIGURES



## Abbreviations

aa: amino acid
bp: base pair
TIL: Tumor infiltrating lymphocytes
ORF: open reading frame
PBMC: peripheral blood mononuclear cells
IRES: internal ribosome entry site
DC: dendritic cell
ITAF: IRES trans-acting factor
LC-MS/MS: liquid chromatography, tandem mass spectrometry
lncRNA: long non coding RNA
MRM: multiple reaction monitoring.

## References

[1] Hoos A (2016). Development of immuno-oncology drugs - from CTLA4 to PD1 to the next generations. Nat rev drug discov.

[2] Labarriere N, Khammari A, Lang F, Dreno B (2011). Is antigen specificity the key to efficient adoptive T-cell therapy?. Immunotherapy.

[3] Godet Y, Moreau-Aubry A, Guilloux Y, Vignard V, Khammari A, Dreno B, Jotereau F, Labarriere N (2008). MELOE-1 is a new antigen overexpressed in melanomas and involved in adoptive T cell transfer efficiency. J Exp Med.

[4] Kung JT, Colognori D, Lee JT (2013). Long noncoding RNAs: past, present, and future. Genetics.

[5] Bobinet M, Vignard V, Florenceau L, Lang F, Labarriere N, Moreau-Aubry A (2013). Overexpression of meloe gene in melanomas is controlled both by specific transcription factors and hypomethylation. PloS one.

[6] Chalopin B, Florenceau L, Fradin D, Labarriere N, Moreau-Aubry A (2015). A lineage-specific methylation pattern controls the transcription of the polycistronic mRNA coding MELOE melanoma antigens. Melanoma res.

[7] Ingolia NT, Lareau LF, Weissman JS (2011). Ribosome profiling of mouse embryonic stem cells reveals the complexity and dynamics of mammalian proteomes. Cell.

[8] Ruiz-Orera J, Messeguer X, Subirana JA, Alba MM (2014). Long non-coding RNAs as a source of new peptides. eLife.

[9] Smith JE, Alvarez-Dominguez JR, Kline N, Huynh NJ, Geisler S, Hu W, Coller J, Baker KE (2014). Translation of small open reading frames within unannotated RNA transcripts in Saccharomyces cerevisiae. Cell rep.

[10] Carbonnelle D, Vignard V, Sehedic D, Moreau-Aubry A, Florenceau L, Charpentier M, Mikulits W, Labarriere N, Lang F (2013). The Melanoma Antigens MELOE-1 and MELOE-2 Are Translated from a Bona Fide Polycistronic mRNA Containing Functional IRES Sequences. Plos One.

[11] Godet Y, Desfrancois J, Vignard V, Schadendorf D, Khammari A, Dreno B, Jotereau F, Labarriere N (2010). Frequent occurrence of high affinity T cells against MELOE-1 makes this antigen an attractive target for melanoma immunotherapy. Eur j immunol.

[12] Bobinet M, Vignard V, Rogel A, Khammari A, Dreno B, Lang F, Labarriere N (2012). MELOE-1 antigen contains multiple HLA class II T cell epitopes recognized by Th1 CD4+ T cells from melanoma patients. PloS one.

[13] Rogel A, Vignard V, Bobinet M, Labarriere N, Lang F (2011). A long peptide from MELOE-1 contains multiple HLA class II T cell epitopes in addition to the HLA-A*0201 epitope: an attractive candidate for melanoma vaccination. Cancer immunol immunother.

[14] Petz M, Kozina D, Huber H, Siwiec T, Seipelt J, Sommergruber W, Mikulits W (2007). The leader region of Laminin B1 mRNA confers cap-independent translation. Nucleic acids res.

[15] Martinuzzi E, Afonso G, Gagnerault MC, Naselli G, Mittag D, Combadiere B, Boitard C, Chaput N, Zitvogel L, Harrison LC, Mallone R (2011). acDCs enhance human antigen-specific T-cell responses. Blood.

[16] Croyal M, Fall F, Ferchaud-Roucher V, Chetiveaux M, Zair Y, Ouguerram K, Krempf M, Nobecourt E (2016). Multiplexed peptide analysis for kinetic measurements of major human apolipoproteins by LC/MS/MS. J lipid res.

[17] Croyal M, Ouguerram K, Passard M, Ferchaud-Roucher V, Chetiveaux M, Billon-Crossouard S, de Gouville AC, Lambert G, Krempf M, Nobecourt E (2015). Effects of Extended-Release Nicotinic Acid on Apolipoprotein (a) Kinetics in Hypertriglyceridemic Patients. Arterioscler thromb vasc biol.

[18] Prensner JR, Chinnaiyan AM (2011). The emergence of lncRNAs in cancer biology. Cancer discov.

[19] Godet Y, Moreau-Aubry A, Mompelat D, Vignard V, Khammari A, Dreno B, Lang F, Jotereau F, Labarriere N (2010). An additional ORF on meloe cDNA encodes a new melanoma antigen, MELOE-2, recognized by melanoma-specific T cells in the HLA-A2 context. Cancer immunol immunother.

[20] Bisio A, Latorre E, Andreotti V, Bressac-de Paillerets B, Harland M, Scarra GB, Ghiorzo P, Spitale RC, Provenzani A, Inga A (2015). The 5′-untranslated region of p16INK4a melanoma tumor suppressor acts as a cellular IRES, controlling mRNA translation under hypoxia through YBX1 binding. Oncotarget.

[21] Dobson T, Chen J, Krushel LA (2013). Dysregulating IRES-dependent translation contributes to overexpression of oncogenic Aurora A Kinase. Mol cancer res.

[22] Faye MD, Holcik M (2015). The role of IRES trans-acting factors in carcinogenesis. Biochim biophys acta.

[23] Petz M, Them N, Huber H, Beug H, Mikulits W (2012). La enhances IRES-mediated translation of laminin B1 during malignant epithelial to mesenchymal transition. Nucleic acids res.

[24] Xiong Z, Liu E, Yan Y, Silver RT, Yang F, Chen IH, Chen Y, Verstovsek S, Wang H, Prchal J, Yang XF (2006). An unconventional antigen translated by a novel internal ribosome entry site elicits antitumor humoral immune reactions. J immunol.

[25] Weingarten-Gabbay S, Elias-Kirma S, Nir R, Gritsenko AA, Stern-Ginossar N, Yakhini Z, Weinberger A, Segal E (2016). Comparative genetics. Systematic discovery of cap-independent translation sequences in human and viral genomes. Science.

