# Supplementary material for: IRES-dependent translation of the long non coding RNA meloe in melanoma cells produces the most immunogenic MELOE antigens
Source: Oncotarget. 2016 Jul 29;7(37):59704–13. doi: 10.18632/oncotarget.10923 (PMC5312342; doi:10.18632/oncotarget.10923)
Supplement: Supplementary file 1 [file oncotarget-07-59704-s001.pdf]

IRES-dependent translation of the long non coding RNA *meloe* in melanoma cells produces the most immunogenic MELOE antigens

Supplementary Materials

```
Atattcatgagaatacagactttttttgttttgatgctgcattctttaaggagagaaggttttattgttttcttcagggt
gctttgggtcaaaagcactagcagcttctgttgggaactcgtggcattttgaATGGTTTTTGATACAGAAATAGCACAA
GTCACGTCTGACACAGCCGTAGGCGCACGAATGCGTGAATGCGATTGTGGTTCTCATCTCTGCAGCTGGTGTGTGGGGC
CGGGCCTGGGACAGCTTGGGCCTCATCCCCACCCACCCGGCTCCCAGGATCCTCATTAGgaagccctcaggagccctgg
ggtgggtctccttgccctgtgaacgctccatttgtgttattgaatccaacataaatgcagcattgtcttcagaggtgag
caatggccgtggccggaggtgcgcatgggatgcaggcggcgtgtgctgctgttgattttctctgctctgctcttagctt
actttagcagatagtggagtttcacgtggcagtccggtaaaccaaggcagtgtgaacatcccttccccacaATGAGTGA
AAATGCAGGAGGTGCCGTAGCGAGAACAGCGACAGCATTCTGCGCATTGGTGAGCCCGACTCCCCAGCCTCGGTGCCCA
CCGAAGCCCCCTCTGGCTGCACTGTGCCAGTGAgtccaggctccttctcagcagaaggtggctgctctgcagggcccct
cgcctccctgctcctcaggggacccgcactcttccatgtccgtagggagacgcgcaccgagctggagacacagatctgc
gaggagtaacttgcctctctcaccagcactgccaggggcggatcagcgccagcaccttccagcatgcgctaaactgctg
gagaagctggagtggctggaaggagccctgcctgcctgccctcctcctcctcagttgctggccttggcagctctagggt
gacccagcaggggcctggaggagtaggaagcagcttcccagctgcagtctgcatcatcctctctgtgctgtcacagaat
ggaaggttttagcagaaaaaggagtttgtaagcgacattcagaagatttgggcagcagtttcttcccccatagggtcct
gtcttttgggagtggggagtgctgggccaggctgctcgctggctgtttcacaggtggtggaatttaagagcagacttgg
gtcgagctgtgaccgttcaggtgatgcacgcacaggaggttcagctgtgtgcctgtgaggcgggcaggagtgtcaccca
agcttcagaagagaattccccggcagcagcgtggcgctcagcatgttccgtgtctgactgttttgattgtctctcagtc
cagttgacaaattccttttaagatgaaatttgaattaatttgcagaacttgtacaaatctccaagaaaacagcttaagg
aacagattaaagaatgaatgccttaggaattaaagcattaatagctcttgagtagactggatggaaagATGAGTTGTGT
AGGTTATCCAGATGAGGCTACATCCAGGGAGCAATTCTTACCTTCAGAGGGCGCTGCCTGTCCTCCTTGGCATCCAAGT
GAGCGCATCTCCTCCACGCTCAATGATGAATGCTGGCCGGCATCCCTGTGAggaaccttgtgatcattttccatttctg
aaaactcccaggtggtgggggcccttggcccacatcagcattgaccaccccgggctcagatctggccaggtgcctgcgt
tgtgatgggaatttcagtacactgaattttgcctctgaccctgatgaaatagcttcggtggcatttgcatcaagatcat
gttagtgtcatctctattagatgctttggagcaaacatgaacttgggtttccttttaagatgtcctgtgattccAGatt
caggggaatctgagaaaagtttgaagaaagaaaattccactcggccagccaaccttgggtgtgcagagcctgccccgcc
ttccccactttgtcctgagaagctgggtcctccccagcaccagagttgctgctgcttcccctTgcgcCcttggctgctc
tcccggccccaagcctgagtgacactctaggattgcagatggcaggctggtctctgggctcccccgtccccatgaccct
ccacctgcacccagacctctgggtccttgctcaggcagcccctgccctcggtgagacgcagcccactgtgggcccttcc
cctgggcctgccatcagctcctgtgctcctcggcgccccagcggctgccctgggcccgttcccacagcacctggcacac
agcaggagccatgaatgttctttgggaactgaatgaggagtggaaatgcgggcatgtcatgtttttgaagttaataaaa
aaaatcccttaaaagttgaaaaaaaaaaaaaaaaaaaaaaaaa
```

Supplementary Figure S1: *meloe* cDNA full length sequence. MELOE-1 (ORF 132–296) (blue), MELOE-2 (ORF 546–665) (green) and MELOE-3 (ORF 1491–1631) (red) are indicated.

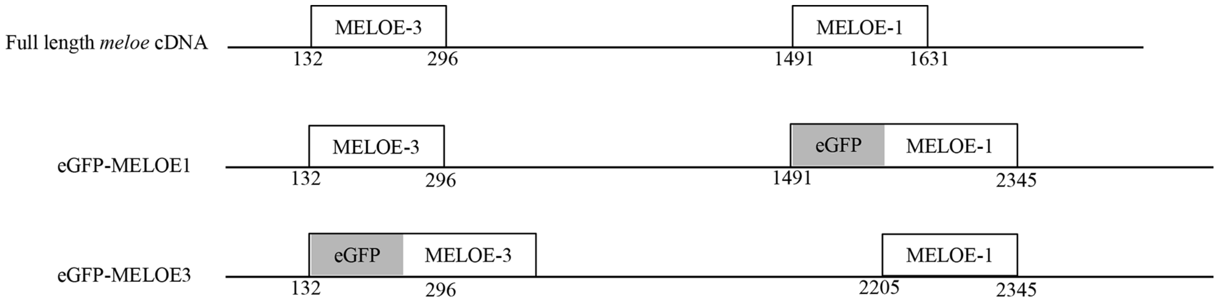

Supplementary Figure S2: Design of *meloe* eGFP-constructs.

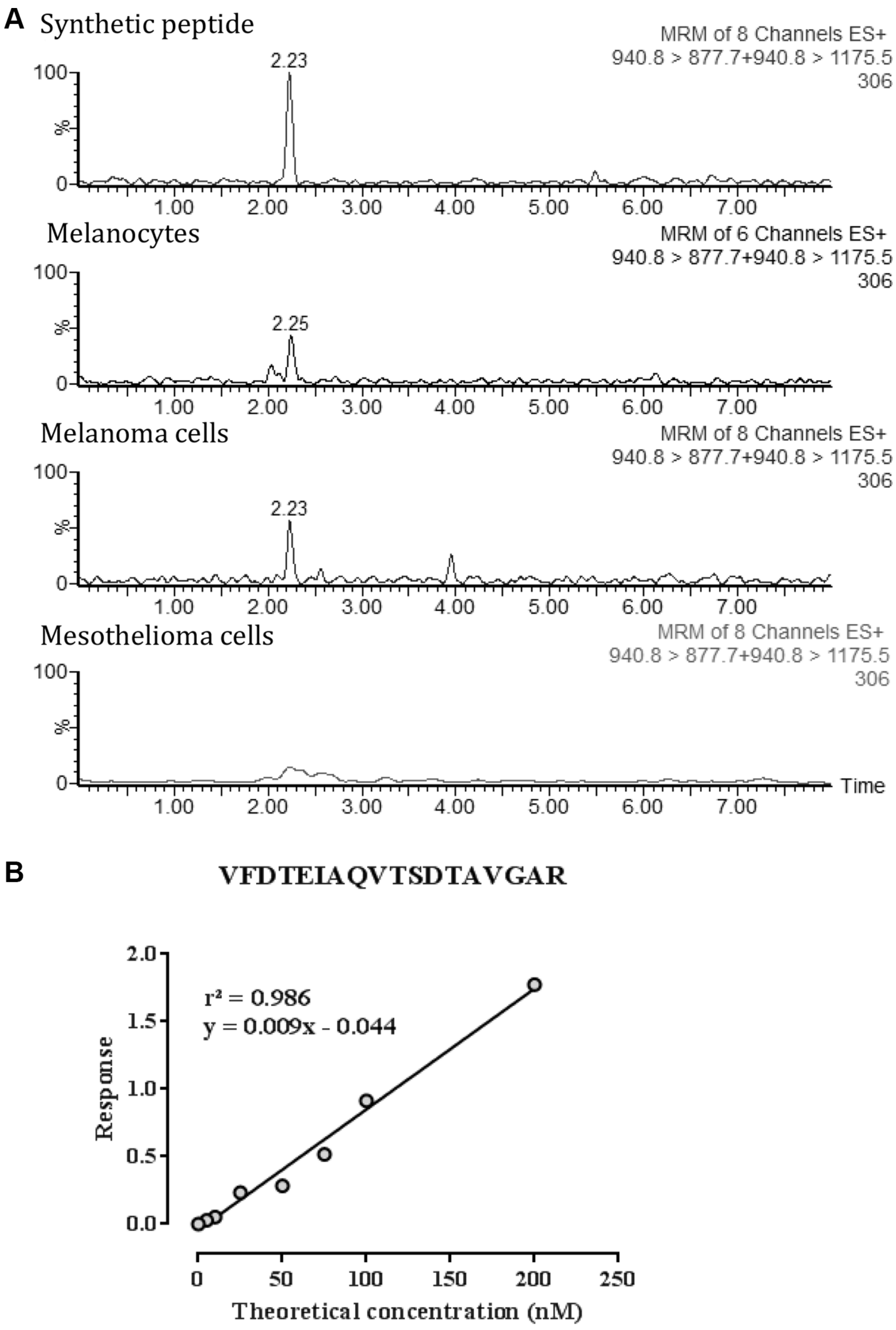

Supplementary Figure S3: LC-MS/MS detection of MELOE-3. (**A**) Representative chromatograms obtained by LC-MS/MS for the synthetic peptide (50 nM) or different cell lines. (**B**) Calibration curve obtained with the synthetic VFDTEIAQVTSDTAVGAR peptide by LC-MS/MS.

MELOE-1

```
MSCVGYPDEATSREQFLPSEGAACPPWHPSERISSTLNDECWPASL
 SCVGYPDEATSREQFLPSEG
          TSREQFLPSEGAACPPWH
                 PSEGAACPPWHPSERISSTL
                         PWHPSERISSTLNDECWPASL
```

MELOE-3

```
MVFDTEIAQVTSDTAVGARMRECDCGSHLCSWCVGPGLGQLGPHPHPPGSQDPH
 VFDTEIAQVTSDTAV
           SDTAVGARMRECDCG
                    RECDCGSHLCSWCVG
                             CSWCVGPGLGQLGPH
```

Supplementary Figure S4: Overlapping peptides from MELOE-1 and MELOE-3. The four peptides of 20aa (MELOE-1) and 15aa (MELOE-3) used to stimulate PBMCs from healthy donors are shown, with potential HLA-A0201 epitopes identified by HLA binding predictions in red.
